# The Magpie Trial: a randomised trial comparing magnesium sulphate with placebo for pre-eclampsia. Outcome for children at 18 months

**DOI:** 10.1111/j.1471-0528.2006.01165.x

**Published:** 2006-12-12

**Authors:** Magpie Trial Follow-Up Study Collaborative Group

**Keywords:** Longterm follow-up, magnesium sulphate, pre-eclampsia, randomised trial

## Abstract

**Objective:**

To assess the long-term effects of *in utero* exposure to magnesium sulphate for children whose mothers had pre-eclampsia.

**Design:**

Assessment at 18 months of age for children whose mothers were recruited to the Magpie Trial (recruitment 1998–2001 ISRCTN 86938761), which compared magnesium sulphate with placebo.

**Setting:**

Follow-up of children born at 125 centres in 19 countries across five continents.

**Population:**

A total of 6922 children were born to women randomised before delivery at follow-up centres. Of these, 2271 were not included for logistic reasons and 168 were excluded (101 at a centre where <20% were contacted, 40 whose death or disability was due to a problem at conception or embryogenesis and 27 whose parent/s opted out). Therefore, 4483 children were included in follow-up, of whom 3283 (73%) were contacted.

**Methods:**

Assessment by questionnaire, with interview and neurodevelopmental testing of selected children.

**Main outcome measures:**

Death or neurosensory disability at age of 18 months.

**Results:**

Of those allocated magnesium sulphate, 245/1635 (15.0%) were dead or had neurosensory disability at 18 months compared with 233/1648 (14.1%) allocated placebo (relative risk [RR] 1.06, 95% CI 0.90–1.25), and of survivors, 19/1409 (1.3%) had neurosensory disability at 18 months compared with 27/1442 (1.9%) (RR 0.72, 95% CI 0.40–1.29). There were no substantial differences in causes of death or in the risk of individual impairments or disabilities.

**Conclusions:**

The lower risk of eclampsia following prophylaxis with magnesium sulphate was not associated with a clear difference in the risk of death or disability for children at 18 months.

## Introduction

Pre-eclampsia complicates 2–8% of pregnancies.^1^ Although outcome is often good, pre-eclampsia is a major cause of morbidity and mortality for women and children.^2^–^4^ Eclampsia, the occurrence of seizures superimposed on pre-eclampsia, is rare but associated with even higher morbidity and mortality,^5^ particularly in developing countries.^3^ Magnesium sulphate is the anticonvulsant of choice for women with eclampsia.^6^–^8^ The Magpie Trial showed that it is also effective for preventing the first eclamptic seizure, without substantive short-term harmful effects on either mother or baby.^9^,^10^

The long-term impact of *in utero* exposure to magnesium sulphate is unclear. Case–control studies have suggested that maternal treatment shortly before birth may lower the risk of cerebral palsy in surviving very-low-birthweight infants.^11^–^13^ A recent randomised trial found some evidence to support this view.^14^ However, trials comparing magnesium sulphate with alternative agents for tocolysis of preterm labour show an increase in the risk of fetal, neonatal or infant death associated with magnesium sulphate,^15^ although these studies used a higher dose (at least double), and gave it for considerably longer, than that in the Magpie Trial.

We contacted women recruited to the Magpie Trial when their children were 18 months or older. The main objective was to determine whether magnesium sulphate for pre-eclampsia affects the child’s chance of surviving without major neurosensory disability.

## Methods

Between 1998 and 2001, 8804 women with pre-eclampsia during pregnancy or labour were recruited to the Magpie Trial at 175 hospitals in 33 countries.^10^ They were randomly allocated to receive either magnesium sulphate or placebo as an intravenous loading dose followed by 24 hours of maintenance therapy. Treatment details have been reported elsewhere.^10^,^16^

**Figure 1:**
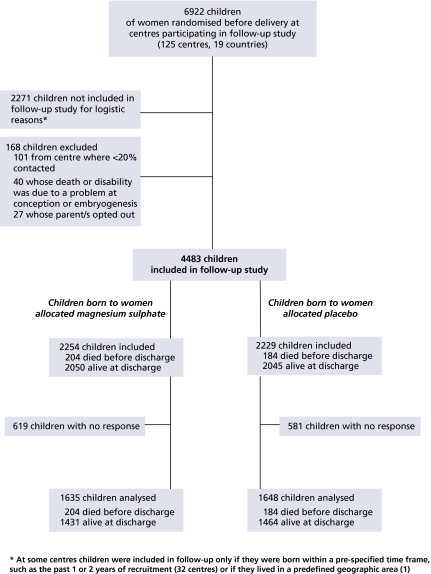
Consort flow for children included in follow up.

These 8804 women gave birth to 9153 children, 9024 of whom were included in our analysis of outcome at discharge from hospital.^10^ Of these, 2102 babies were never eligible for follow-up; 127 who were unlikely to have been alive at trial entry (fetal heart beat not heard at trial entry and macerated stillbirth within 24 hours) and 1975 born at 50 centres, predominantly in developing countries, where follow-up was not thought possible either because reliable contact details were not available at discharge from hospital or because there was no local coordinator for the study. Children who ultimately participated in this follow-up study, having been born to the cohort of women recruited before delivery at the 125 centres in 19 countries participating in follow-up, are shown in Figure 1. All recruited children were included in follow-up at 92 centres (2145 children) and at least 90% at 106 centres (3330). Everyone involved in tracing and assessment remained blind to the allocated treatment. The protocol for follow-up is available elsewhere.^17^ Outcome for women^18^ and narrative accounts of the collaborators^19^ are published separately.

### Ethics approval and consent

All hospitals secured local research ethics committee approval before starting recruitment to the main trial. Women were informed that they might be contacted for follow-up prior to giving consent for recruitment. Therefore, some centres did not require that the follow-up study be resubmitted to an ethics committee. Others required a new submission or considered the follow-up protocol as an amendment to the original trial protocol.

### How children were assessed

Children were screened using the Ages and Stage Questionnaires (ASQs).^20^ Thirty questions cover the five domains: communication, gross motor, fine motor, problem solving and personal–social. To pass, the child has to pass all five domains. An additional section addresses general parental concerns, and it is not scored. We added two questions about use of health service resources.^17^ Up to 24 months, age is adjusted for gestation at birth. Each questionnaire is valid for 4 weeks either side of the target age. This was extended by an additional week for each year of the child’s age.

For centres where the full ASQ was not thought feasible for cultural or language reasons, we shortened the questionnaires by selecting the questions considered most likely to predict severe developmental delay. Three questions were selected in each of the gross motor, communication and problem solving domains, together with five general questions.^17^ To pass, the child had to have at least one ‘yes’ in each domain.

Questionnaires were available in English and Spanish. They were sent by post, administered in clinic (usually a clinic provided for children within the study) or during a home visit, or completed over the telephone. If the family could not be contacted directly, information about whether the child was ‘alive and well’ was collected, whenever possible. The aim was to assess children when they were at least 18 months, but this varied depending upon what was feasible.

Children who passed the ASQ for their own age, or for an older age group, were considered screen negative. Children who failed the ASQ were considered screen positive. Also considered screen positive were children whose ASQ was incomplete and could not be scored and those who passed the ASQ for children in a younger age group. Screen-positive children, and a sample of screen-negative children, were invited for a clinical and neurodevelopmental assessment using the Bayley Scales of Infant Development (BSID-II)^21^ or the Griffiths Tests,^22^ or an alternative test if neither was available. If no test was possible, we relied on clinical history and examination, including information about non-neurosensory disability (see below) using the Health Status Questionnaire.^23^ The person doing this assessment was usually aware of whether or not the child had passed the ASQ.

In the UK, the Office for National Statistics provided the date and cause for all deaths. When surviving UK children were at least 18 months old, a questionnaire was also sent to their GP asking about neurosensory function and general health.

### Data review

Data for each child who died, or was thought might have disability, were reviewed by an independent paediatrician. A second paediatrician reviewed a 10% random sample of these, plus any with uncertainty about the diagnosis. Outcome was determined by consensus. Data for a 20% sample of children considered screen negative were also reviewed, together with all those who had a short ASQ only.

### Outcome measures

The primary outcome for the follow-up study was the composite measure of death or neurosensory disability at age of 18 months.^17^ Secondary outcomes for the children were death alone, each individual measure of neurosensory disability alone, delayed speech, other disability and contact with health services.

### Definitions of outcome measures for the children

#### Death or neurosensory disability at 18 months of age (primary outcome)

The time point of at least 18 months (corrected for gestation at birth) provided the best balance between feasibility of follow up and certainty of diagnosis. Excluded were (a) cases where death or neurosensory disability was judged to have had its origin at conception or during embryogenesis, (b) deaths after 18 months and (c) disability clearly caused by some event occurring after 18 months. Four children were classified as fulfilling the criteria for inclusion in the composite primary outcome because, although only 12–17 months old when last seen, they were clearly disabled and would be dead or disabled at 18 months (magnesium sulphate, *n* = 3; placebo, *n* = 1).

Children were classified as having neurosensory disability if they were functionally blind (binocular visual acuity <6/60) or deaf (severe enough to need a hearing aid), had severe cerebral palsy (not walking or unlikely to walk unaided by 24 months^14^) or had a developmental quotient (DQ) <−2 SD. Children with a DQ <−2 SD were classified as having definite delay, as were children whose developmental progress was less than that would be expected of an average child half that age. When development had only been assessed clinically or using the Denver Developmental Screening Test-2 (DDST-II)^24^ or its derivative the Lejarraga and Krupitzky test,^25^ children were classed as being ‘likely’ to have developmental delay if progress in at least one domain was clearly less than that was to be expected of a child two-thirds that age. Of the 1288 children who had neurodevelopmental assessment, this was the Lejarraga and Krupitzky^25^ for 743, BSID-II^21^ for 367, DDST-II^24^ for 49, Griffiths^22^ for 46, Baroda (a derivative of the BSID-II)^26^ for 14 and other tests for 69.

#### Other disability

Children with non-neurosensory disability alone (such as needing continuous supplemental oxygen, breathing support, renal dialysis, frequent seizures despite treatment and tube or parenteral feeding)^23^ were classified as having ‘other disability’. So too were children whose cerebral palsy was judged to be not severe.

#### Isolated speech delay

Children with a vocabulary of less than ten words at 24 corrected months but no other developmental problem^23^ were classified as having isolated speech delay, as were older children with equivalent degrees of speech delay.

### Power of the study

We anticipated that 2680–3210 children whose mothers were randomised before delivery would be eligible. We estimated the composite primary outcome (death or neurosensory disability) might affect 20–25% of children in the placebo group. After adjustment for expected losses to follow up, it was anticipated that there would be data on death or disability for 2370–2810 children. This would give 70–80% power (alpha 0.05) to detect a relative difference between the groups of 20% in the primary outcome.^17^

### Statistical analyses

Analyses were based on the groups to which the women and children had been allocated at trial entry. Centres able to contact <20% of included families were excluded. Where appropriate, results are presented as relative risk (RR) with 95% confidence intervals (CI).

Sensitivity analyses for the composite primary outcome were pre-specified^17^ as: excluding children classified as having ‘likely disability’, including children whose death or neurosensory disability originated during conception or embryogenesis, excluding centres where <90% of randomised children were selected for follow up or where selection was not based on a time frame and including centres where 80% or more of the children could not be contacted.

Planned subgroup analyses^17^ were based on the following: severity of the mother’s pre-eclampsia at trial entry (severe, moderate, mild), gestation at birth (≤33 completed weeks, >33 completed weeks), perinatal mortality (PNM) in the country (high, medium or low)^27^ and whether the mother received maintenance therapy of trial treatment by the intramuscular or the intravenous route. In addition, a *post hoc* Poisson regression was conducted for the primary outcome adjusting for gestation at birth, whether the mother had an anticonvulsant before trial entry, PNM in country, route of maintenance therapy, sex of the baby, multiple pregnancy and whether admitted to a special care baby unit.

The analysis of children’s death alone included all deaths and took account of the age at death, using log-rank survival analysis. Cause of death for the babies was classified using standard criteria.^28^,^29^

## Results

Overall, 125 centres in 19 countries in Africa, Asia, the Americas, Australia and Europe participated in follow-up. The coordinating centre in Oxford traced children from the 67 UK centres. Local collaborators traced all other children. Data collection closed on 31 December 2003. For 27 children, the parent/s or carers opted out of follow up. Of these, 16 were ‘alive and well’ when contacted. There is no information on the other 11 children. Also excluded from the analyses were 40 children who died (*n* = 26) or had disability due to a problem originating at conception or embryogenesis (14). Two of these surviving children also had cerebral palsy.

Included in the follow-up study were 4483 children whose mothers were randomised before birth (magnesium sulphate, *n* = 2254; placebo, *n* = 2229) (Figure 1), of whom 388 (9%) were stillborn or died before discharge from hospital and 4095 (91%) were alive at discharge. The main substantive differences between children included in follow-up and those in the trial overall were that a higher proportion came from low or middle PNM countries (61% in follow up versus 43% in the trial overall). Therefore, most of their mothers received the intravenous maintenance regimen for magnesium sulphate (63% versus 52%).^10^ Also, fewer children included in follow-up were born before 33 completed weeks (23% versus 27%).

### Completeness and review of data

Data for 3283 children (73%) were available for analysis (magnesium sulphate, *n* = 1635; placebo, *n* = 1648) (Figure 1). For 106 children in the UK, information was from the GP only. In the UK, 98% of children were contacted. Outside the UK, 11 centres contacted all included children and 36 contacted more than half of included children. Data were available for 57% of children in high PNM countries (994/1744), 75% in middle PNM countries (1213/1609) and 95% in low PNM countries (1076/1130) (Table 1). Outcome at discharge from hospital was similar for children included and for those contacted (Table 1).

**Table 1:** Characteristics at trial entry, exposure to magnesium sulphate and outcome at discharge from hospital for children included in follow-up and those contacted

	Children included in follow-up		Children contacted	
		
	MgSO4, *n* = 2254	Placebo, *n* = 2229	MgSO4, *n* = 1635	Placebo, *n* = 1648
**Mothers’ characteristics at trial entry**				
Singleton pregnancy	2094 (93)	2096 (94)	1522 (93)	1554 (94)
Pre-eclampsia				
Severe	526 (23)	553 (25)	395 (24)	421 (26)
Moderate	1047 (47)	990 (44)	721 (44)	709 (43)
Mild	691 (30)	686 (31)	519 (32)	518 (31)
Prior anticonvulsant	160 (7)	167 (7)	110 (7)	110 (7)
≤33 completed weeks of gestation	529 (23)	522 (24)	398 (24)	396 (24)
Intravenous maintenance regimen	1429 (63)	1391 (61)	1142 (70)	1118 (68)
High PNM country	864 (38)	880 (40)	479 (29)	515 (32)
Middle PNM country	823 (37)	786 (35)	617 (38)	596 (36)
Low PNM country	567 (25)	563 (25)	539 (33)	537 (33)
**After trial entry**				
Exposure to MgSO_4_				
None	85 (4)	2216 (99)	70 (4)	1638 (99)
Median (IQR) (g)	14 (5–29)	0	18 (9–29)	0
Time to delivery, median (IQR) (hours)	12 (4–42)	11 (4–39)	12 (4–46)	11 (4–46)
**Outcome at discharge from hospital**				
Born ≤33 completed weeks	435 (19)	418 (19)	331 (20)	326 (20)
Stillbirth or died before discharge	204 (9)	184 (8)	204 (12)	184 (11)
In special care baby unit*	804 (38)	773 (37)	576 (38)	556 (36)

IQR, interquartile range.

Data are *n* (%) unless otherwise indicated.

* Liveborn babies only: selected children in MgSO_4_ group, *n* = 2132, and in placebo group, *n* = 2108; contacted children in MgSO_4_ group, *n* = 1513, and in placebo group, *n* = 1527.

There was 88% agreement on the data review, with all discrepancies resolved by discussion. For 73 children, the neurodevelopmental test was overruled as having been incorrectly applied (magnesium sulphate, *n* = 34 and placebo, *n* = 39).

### ASQ performance

Of the 2610 children for whom an ASQ was completed, 86% (2233) had at least one long ASQ and 15% (377) had the short ASQ only (Table 2). Overall, 636 (24%) were considered screen positive, of whom 549 (86%) had further assessment (Table 3). How well the full ASQ distinguished between children likely to have or not to have neurosensory disability is summarised in Table 4. No children passed the ASQ and were later found to have neurosensory disability among either the 377 screened with the short ASQ only or the 215 screened with the full ASQ when aged younger than 18 months. Data were reviewed for 436 children who passed the full ASQ; 122 were reviewed because additional information in the general questions or elsewhere suggested that they might have disability and 314 randomly selected from the remainder, three of whom had neurosensory disability.

**Table 2:** For children alive at discharge from hospital and traced: information from tracing and screening

	MgSO_4_, *n* = 1431	Placebo, *n* = 1464
**ASQs completed**	1283 (90)	1327 (91)
At least one full ASQ	1101	1132
Short ASQ only	182	195
**No ASQ**	148 (10)	137 (9)
Information from GP questionnaire (UK)	59	47
‘Alive and well’ only information	63	63
Child dead	24	25
Paediatric assessment but no ASQ	2	2
**ASQ scoring**		
Complete	1186 (83)	1186 (81)
Ratio score for full ASQ	87 (6)	128 (9)
Incomplete and unable to score	10 (1)	13 (1)
**When ASQ completed**		
Within correct time frame*	1070 (75)	1049 (72)
Outside correct time frame	213 (15)	278 (19)
ASQ for an older age group	131	162
ASQ for a younger age group	82	116
**Failed ASQ**	240 (17)	226 (16)
ASQ for correct age or an older age group	229	209
ASQ for a younger child	11	17

Data are *n* (%).

* Based on Magpie Trial time frame.^17^

**Table 3:** For children alive at discharge from hospital: further assessment after tracing and screening

	MgSO4, *n* = 1431	Placebo, *n* = 1464
**Outcome determined without further assessment**	25 (2)	26 (2)
Known severe neurosensory disability	1	1
Child died after discharge	24	25
Screen-positive children*	311 (22)	325 (22)
Clinical assessment + neurodevelopmental test	217	215
Clinical assessment alone	51	66
ASQ only, no further assessment	43	44
Screening to assessment (days), median (IQR)	5 (0–55)	3 (0–52)
Screen-negative children**	972 (69)	1002 (68)
Clinical assessment + neurodevelopmental test	413	440
Clinical assessment alone	245	241
ASQ only, no further assessment	314	321
Screening to assessment (days): median (IQR)	0 (0–6)	0 (0–4)
**No ASQ but had neurodevelopmental test**	2	1
**Child’s age at assessment or when known to be ‘alive and well’**		
<12 months	20	31
12–17 months	195	135
18–23 months	219	259
≥24 months	914	967
Data from GP only	59	47
**Data reviewed by paediatrician†**	685 (48)	739 (50)
Considered screen-positive	311	326
Considered screen-negative	355	397
Died after discharge	24	25
No ASQ	19	16

IQR, interquartile range.

Data are *n* (%) unless otherwise indicated.

* Children who failed the ASQ regardless of whether or not within the correct time frame, or who passed it but the ASQ was for a younger age group or whose ASQ could not be scored.

** Children who passed the ASQ for their correct age or for an older age group.

† Data also reviewed for the 388 children who died before discharge.

**Table 4:** For children with a full ASQ: screening result and whether the child had neurosensory disability

ASQ outcome	Neurosensory disability		
	
	Yes*	No	Total
Failed**	42	538	580
Passed	3***	433	436
Total	45	971	1116

* Includes likely neurosensory disability.

** Includes 129 who passed the ASQ for a younger age group, 26 who failed the ASQ for a younger age group and 20 whose ASQ could not be scored. Excluding those with an ASQ for a younger age group, the negative predictive value is 433/436 = 99.3%.

*** All were ≥18 months when screened; two passed scored section of the ASQ, but there was concern about hearing or speech in general section.

### Outcome for the children

**Figure 2:**
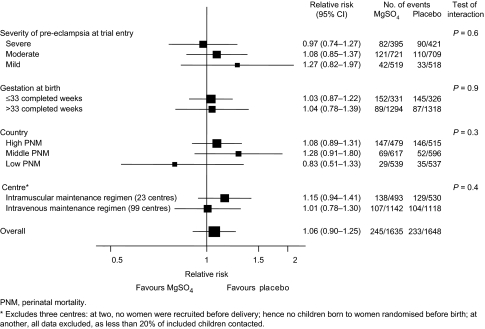
Effects of treatment on death or neurosensory disability at the age of 18 months: subgroup analyses.

Of the children whose mothers were allocated magnesium sulphate, 245/1635 (15.0%) had the primary outcome of death or noncongenital neurosensory disability compared with 233/1648 (14.1%) allocated placebo (RR 1.06, 95% CI 0.90–1.25) (Table 5). This result was consistent across the pre-specified subgroups (Figure 2), and it was not substantially altered by any pre-specified sensitivity analysis (data not shown) or by adjusting for major prognostic factors (RR 1.07, 95% CI 0.92–1.24) or after excluding children seen only when younger than 18 months (RR 1.10, 95% CI 0.93–1.29).

**Table 5:** For all selected children: death or neurosensory disability

	MgSO4, *n* = 1635	Placebo, *n* = 1648
**Death after randomisation and ≤18 months**	226 (13.8)	206 (12.5)
Stillbirth or died before discharge	204	184
Died after discharge	22	22
Neurosensory disability*	13 (0.8)	19 (1.2)
Blind	3	3
Deaf	2	1
Severe cerebral palsy	3	9
Developmental delay	11	15
**Likely neurosensory disability**	6 (0.4)	8 (0.5)
Blind	—	—
Deaf	1	—
Severe cerebral palsy	—	—
Developmental delay	5	8
**Death or neurosensory disability at 18 months**		
For all contacted children	245 (15.0)	233 (14.1)
For children followed until either they developed the primary outcome or at least 18 months old**	245 (17.2)	233 (15.7)
**Other significant disability**	3 (0.2)	5 (0.3)
Other cerebral palsy not included above	2	1
Other disability	—	3***
**Isolated speech delay**	23 (1.4)	29 (1.8)
Simple, probably transient	22	27
Features suggestive of autism	1	2

Data are *n* (%).

* Some children had more than one disability. One child with known neurosensory disability did not have an ASQ.

** MgSO_4_, *n* = 1421; placebo, *n* = 1480.

*** One child had each of the following: chronic oxygen dependency at 18 months following viral chest infection at 3 months, apraxia, Erbs palsy.

There was no significant difference between the groups in the risk of neurosensory disability at 18 months (19/1409 versus 27/1442; RR 0.72, 95% CI 0.40–1.29). Fifteen children were identified as having cerebral palsy (5 versus 10); this was severe for 12 (3 versus 9). Two were from a country with high PNM, seven with middle PNM and six with low PNM.

Of the 2895 children alive at discharge and traced, 44 (1.7%) died after discharge; there were no substantial differences in causes of death (Table 6). In total, 226/1635 (13.8%) of children in the magnesium group were stillborn or died compared with 206/1648 (12.5%) allocated placebo (RR 1.11, 95% CI 0.93–1.32) (Table 6).

**Table 6:** For all children selected and traced: death and cause of death

	MgSO_4_, *n* = 1635	Placebo, *n* = 1648
**Included in primary outcome (died ≤18 months)**	226 (13.8)	206 (12.5)
Stillbirth	122	121
Liveborn		
Died before discharge	82	63
Died after discharge	22	22
**Not included in primary outcome**		
Died >18 months	2	3
**Total deaths**	228 (13.9)	209 (12.7)
**Cause of death**		
Infection before/during birth	2	1
Antepartum stillbirth*	122	120
Preterm birth	56	48
Intrapartum asphyxia/trauma	20	13
Infection after birth	15	14
External agent after birth	1	2
Sudden infant death	1	1
Unclassifiable	11	10

Data are *n* (%).

* Does not include infection acquired before or during birth.

Of the children for whom we had either a completed ASQ or information from their GP, more than half were reported to have attended a clinic since discharge from hospital after delivery (759/1342 versus 729/1374) and almost one-quarter had been admitted to hospital (292/1342 versus 301/1374). There were no substantial differences in these outcomes between the treatment groups. The most common reason for hospital admission was respiratory problems (106 versus 109).

## Discussion

Our earlier report showed that magnesium sulphate is effective for prevention of eclampsia.^10^ Results presented here provide reassurance about safety for the children in the longer term and are generalisable to both developed and developing countries. Magnesium sulphate for pre-eclampsia was not associated with any substantive reduction in survival without neurosensory disability for the children exposed while *in utero*.

When the Magpie Trial was designed, follow-up was planned in the UK only. Once it became clear that more than 80% of recruitment was from developing countries, it seemed imperative to at least try to contact a sample of these women and children. Although 50 centres were unable to participate in follow-up, this could not have introduced bias into the assessment of outcome, as randomisation had been stratified by centre. For the 125 centres that did participate in follow-up, 74% included follow up of all children recruited and 85% included at least 90% of children. The decision about who was included in follow-up was made at the start of the study, blind to treatment allocation. The primary reason why some centres only tried to contact a proportion of children was that many of those born early in the trial would have changed address, possibly several times, and so would be impossible to trace. Hence, in these centres, children were only included in follow up if they were born after a pre-specified date. At one centre, an additional factor was that it was unsafe to visit certain geographic areas, so those with a discharge address within these pre-specified areas were not included. It is implausible that these factors could have been related to outcome, and as they are likely to be equally distributed between the treatment groups because of the randomisation, it is unlikely that they introduced any bias.

Having provided reassurance that there was no potential bias between the groups in the way children were selected for inclusion in follow-up, our pragmatic philosophy was that any information about outcome was better than none. This approach enabled us to trace families and assess children in situations where follow-up had previously been thought impossible. We are not aware of other perinatal trials that have conducted follow-up in a comparable range of countries and settings. Restricting the study to centres able to guarantee that a high proportion of children could be traced would have excluded most low-income countries. Overall, 73% of included children were contacted, which is a remarkable achievement. In many countries, there was a strong impression that women valued this continued interest in the welfare of their child. The chief exception was some rural communities, where strangers visiting a remote village might be regarded with suspicion.

A key challenge was to identify an appropriate and simple tool for screening large numbers of young children over a wide age range and in a variety of settings and countries. The ASQ^20^ seemed to meet these criteria. Although it is user friendly, with pictures and clear text, each questionnaire is three pages. As this was initially thought to be rather long for some communities, especially those who were non-English speaking and predominantly rural, we shortened it to one page. In practice, many centres found administering the full ASQ less problematic than anticipated. Uptake of the short ASQ was therefore lower than expected.

The use of a variety of tools for neurodevelopmental assessment and that it was not possible for all children to have a full assessment were limitations of this study. Also, this follow-up has less power than the original trial as it was not feasible to include all children randomised, those included tended to be at lower risk of adverse outcome and it was harder to contact families in high PNM countries. These losses to follow-up do not appear to have had a substantive impact, however, as results are relatively consistent across countries with differing completeness of follow-up. In a blinded trial such as ours, it is unlikely that not being able to trace 27% of children after discharge would introduce appreciable bias in the conclusions, as the reasons for failure to contact them will have been equally true of children in both arms of the study. As those who were followed-up had been randomly exposed to magnesium sulphate, these data still allow an unbiased assessment of the treatment effect of antenatal magnesium sulphate on longer term outcomes. Moreover, the total numbers eventually followed, 2895 survivors from 3283 enrolled, are large relative to other antenatal trials and still allow power to detect uncommon adverse outcomes. By comparison, the Cochrane review of antenatal steroid therapy,^30^ the most obvious example of an antenatal intervention that has improved outcome for preterm infants, comprises data on only 3517 fetuses exposed, of whom 3184 survived and on whom long-term outcome data are reported for just 778 children.

Magnesium sulphate given as neuroprotection to women at risk of preterm birth before 30 weeks of gestation has been evaluated in one previous trial;^14^ there was a tendency to a lower risk of death or cerebral palsy at 2 years if the mothers were allocated magnesium sulphate rather than placebo (RR 0.83, 95% CI 0.66–1.03). Nearly 8% of surviving babies in that study had cerebral palsy; only 17 surviving children were identified as having cerebral palsy in the present study, and in two of these, the problem was thought to have arisen during conception or embryogenesis. However, 10 of the other 15 children were among those whose mothers were allocated placebo (Table 5). The numbers involved are small, and this imbalance could have arisen by chance, but the trend is in the same direction as in the earlier trial.^14^ In our study, only one baby with cerebral palsy and 2.3% of all the babies were born before 30 weeks of gestation.

## Conclusions

Magnesium sulphate for women with pre-eclampsia more than halves the risk of eclampsia (RR 0.42, 95% CI 0.29–0.60) and probably reduces the risk of maternal death before discharge from hospital (RR 0.55, 95% CI 0.26–1.14) compared with placebo. No substantive harmful effects were apparent in the short term, for either mother or baby. Exposure to magnesium sulphate while *in utero* was not associated with a clear difference in the risk of death or disability for children at 18 months. These data provide reasonable reassurance about the long-term safety of magnesium sulphate for the children, at the dosage used in our study.

## Magpie trial follow-up study collaborative group

### Central coordination and coordinating team for UK

Lelia Duley (clinical coordinator), Barbara Farrell (trial manager), Nina Armstrong (follow-up study coordinator), Patsy Spark (trial programmer), Barbara Roberts (trial secretary), Rebecca Smyth (research midwife), Mary Tivnan (data manager), Anna Laws (research assistant), Noelene Corfield (data assistant), Annette Salter (data assistant) and Lisa Thorn (data assistant).

### Statistical support

Douglas Altman and Ly-Mee Yu of Centre for Statistics in Medicine and Patsy Spark of Central coordinating team.

### Coordinating team for Argentina

Edgardo Abalos (clinical coordinator), Berenise Carroli (coordinator), Lucrecia Dellepiane (field worker), Marina Duarte (paediatrician), Hebe Fernandez (field worker) and Daniel Giordano (programmer).

### Management group

Douglas Altman, Nina Armstrong, Mike Clarke, Lelia Duley, Barbara Farrell (chair), Alastair Gray, Edmund Hey, James Neilson, Judit Simon, Patsy Spark, Rebecca Smyth and Ly-Mee Yu.

### Paediatric review

Lex Doyle and Edmund Hey.

#### Assessment advisors

Tom Kelly (Newcastle, UK) and Jane Squires (Oregon, USA).

### Steering committee

Douglas Altman, Rory Collins, LeliA Duley (observer), Barbara Farrell (observer), Anna Karaoglou (observer), Richard Lilford, Jack Moodley, James Neilson (observer), Stephen Robson, Ian Roberts, Peter Rubin (chair), James Thornton, Sara Twaddle, José Villar (observer), Isabel Walker and Christopher Watkins (observer).

### Writing committee

Edgardo Abalos, Douglas Altman, Nina Armstrong, Lex Doyle, Lelia Duley (chair), Barbara Farrell, Edmund Hey, Jack Moodley, James Neilson, Patsy Spark, Rebecca Smyth and Ly-Mee Yu.

### Collaborators by country

#### Albania

Maternity Hospital No. 2: A Bimbashi, E Demalia, O Gliozheni and A Shpata.

#### Argentina

Hospital Carlos A Durand: A Karolinski, M Lamas, M Pesaresi and V Wainer; Hospital Del Centenario: W Barbato; Hospital Eva Perón: M Paciocco; Hospital Interzonal Dr José Penna: M Bertin, E Boiza, J Castaldi and Y Partida; Hospital J B Iturraspe and Hospital Jose Maria Cullen: C Arias, M Farri and G Kerz; Hospital Juan R Vidal: J Aguirre, M de Sagastizábal, R Falcone and E Morales; Hospital Maternidad Martin: G Carroli; Hospital Nacional Prof. Alejandro Posadas: S Krupitzky, S López, M Palermo and D Montes Varela; Hospital Provincial Santa Fé: H Delprato; Hospital Regional ‘Dr Ramón Carrillo’: H Camusso and M Curioni; Hospital Roque Saénz Peña: E Ludmer; Luis Carlos Lagomaggiore: R Brandi, R Martin, W Mesas and R Taralli; Maternidad Provincial Salta (Nuevo del Milagro): M Lezaola and M Morosini; Materno Infantil ‘Ramón Sardá’: E Andina, L Bernal, M Estiú and E Ulens; Materno Provincial Córdoba: B Ortiz de Speranza and A Peyrano; Zonal de Agudos ‘Héroes de Malvinas’: M Damiano and C Saumench.

#### Australia

Royal Brisbane & Women’s Hospital Perinatal Research Centre: J Horn, M Pritchard, V Smith-Orr and M Wilson; The Townsville Hospital: A Lawrence and D Watson; Women’s and Children’s Hospital: C Crowther and J Paynter.

#### Bangladesh

Bangabandhu Sheikh Mujib Medical University: Ashrafunnessa, M Mannan, M Shahidullah and L Shamsuddin.

#### Brazil

Hospital das Clinicas Da UFPE: C Barros Santos, S Freire and E Melo.

#### Colombia

Fundaciòn Clínica Valle del Lili: E Cobo and M Jaramillo; Universidad Nacional de Colombia, Hospital Materno Infantil de Bogotá: C Cardozo, N Fandiño, H Gaitán and L Montaño; Hospital Universitario de San Ignacio: J Lozano and M Rojas.

#### Cuba

Docente Gineco-Obstétrico ‘Eusebio Hernández’: A Breto Garcíá, A Fuentes Ramirez, R Garcíá Mírás and S Sampera; Hospital Gineco-Obstétrico ‘América Arias’: U Farnot, E Gómez, G Rojas and R Valdés.

#### Egypt

Assiut University Hospital: H Abd El-Kreem, T Al-Hussaini and E Hammad.

#### Ghana

Komfo Anokye Teaching Hospital: K Danso, E Kwapong and F Ofosu-Barko.

#### India

Christian Medical College Hospital: M Padmini Jasper, A Peedicayil and A Regi; J N Medical College AMU: R Sharma; King Edward VII Memorial Hospital: A Chauhan, V Raut and R Udani; Maulana Azad Medical College and Loknayak Hospital: S Batra, A Muthal-Rathore, S Ramji and V Zutshi; SAT Hospital, Medical College Trivandrum: S Balakrishnan, E Eapen and G Koshy; Shree Maharani Shantadevi Hospital: B Ambardar, P Vadakkepat and D Vaidya.

#### Malawi

Queen Elizabeth Central Hospital: V Lema, Y Rijken and E Tadesse.

#### Nigeria

Centre for Research in Reproductive Health (coordination of hospitals in Sagamu region): O Dada and A Sofekun; Nigerian Security Printing and Minting Staff Hospital: C Ohiaeri; Olabisi Onabanjo University Teaching Hospital: T Runsewe-Abiodun; University College Hospital: I Adewole, A Adeyemo, B Brown and R Oladokun; University of Port Harcourt: O Adewale, N Inimgba, C John and R Ogu; Usmanu Danfodiyo University Teaching Hospital: B Ekele, A Isah and B Onankpa.

#### Pakistan

Jinnah Post Graduate Medical Centre: R Jamelle and D Junejo; Lady Reading Hospital: N Ruby Faiz, F Gul and A Sherin; Maternal and Child Health Centre: K Bangash, G Mahmud, K Masud and N Tasneem.

#### Sierra Leone

Princess Christian Maternity Hospital: S Gassama and A Soyei.

#### Singapore

KK Women’s and Children’s Hospital: P Agarwal and V Rajadurai.

#### South Africa

Chris Hani Baragwanath Hospital: N Pirani; University of Pretoria (Kalafong Hospital and Pretoria Academic Hospital): S Delport, P Macdonald, R Mokhondo, R Pattinson and M Zondo; King Edward VIII Hospital: M Adhikari, N Mnguni and J Moodley; Tygerberg Hospital: M Carstens, G Kirsten, W Steyn and J van Zyl.

#### USA

OHSU Hospital: A Helwig, S-L Jacobson and R Panosche.

#### Zimbabwe

Harare Hospital: E Hammond and L Masanganise.
